# Dupilumab combined with corticosteroid therapy for Kimura disease with multiple systemic masses: a case report and literature review

**DOI:** 10.3389/fimmu.2024.1492547

**Published:** 2024-10-24

**Authors:** Yansi Lyu, Yaqian Cui, Li Ma, Lvxin Guan, Ziping Wen, Jingkai Huang, Minglan Shi, Suchun Hou

**Affiliations:** ^1^ Department of Dermatology, Shenzhen University General Hospital, Shenzhen, Shenzhen, China; ^2^ Longhua District People’s Hospital, Shenzhen, China; ^3^ Department of Burn and Plastic Surgery, the First Affiliated Hospital of Shenzhen University, Shenzhen, China

**Keywords:** dupilumab, corticosteroid, combination therapy, Kimura disease, multiple systemic masses

## Abstract

To date, the pathogenesis of Kimura’s disease remains unclear, there is no unified diagnostic criterion, the clinical phenotype shows considerable heterogeneity, and there is a lack of optimal treatment strategies. Due to its rarity, treatment strategies for KD are still under exploration. This paper reports a case of a 37-year-old Chinese female presenting with generalized erythematous papules and pruritic eruptions for 12 years, followed by the onset of limb swellings 3 years later, ultimately diagnosed as Kimura’s disease. Considering the patient’s multiple lymphadenopathies and limb swellings with concurrent atopic dermatitis, the treatment regimen included initial dupilumab dosage of 600 mg (300 mg administered in two injections), followed by subcutaneous injections of 300 mg every two weeks for four months. Concurrent oral corticosteroid therapy (methylprednisolone, initial dose 16 mg/kg/day, gradually tapered with tumor regression) was also administered. Following treatment, the patient did not experience severe adverse effects, and the multiple nodules markedly decreased in size. Additionally, serum IgE levels, eosinophil, and basophil counts showed significant reductions. These results demonstrate the significant efficacy of dupilumab combined with oral corticosteroids in treating Kimura’s disease with concurrent atopic dermatitis.

## 1 Introduction

Kimura disease (KD), also known as eosinophilic hyperplastic lymphogranuloma, is a rare chronic inflammatory condition (1). KD predominantly affects young Asian males aged 20 to 40 years, primarily involving the deep subcutaneous regions of the head and neck, particularly the salivary glands, parotid glands, and adjacent lymph nodes (2, 3). Laboratory investigations typically reveal peripheral blood eosinophilia and elevated serum IgE levels (4). Biopsies of lymph nodes and lesions indicate lymphoid tissue hyperplasia, well-developed lymphoid follicles, and a marked increase in eosinophil infiltration (5). Furthermore, the pathological features of KD may include eosinophilic abscesses, capillary proliferation, and perilesional fibrosis (6). Studies have indicated that 59-78% of patients with KD develop nephrotic syndrome, predominantly characterized by membranous nephropathy. This condition often presents with proteinuria, which may occur with or without renal impairment (7, 8). Additionally, KD can be concurrently associated with other conditions such as eczema (9), asthma (10), ulcerative colitis (11), and vasculitis (12).

The etiology and pathogenesis of KD remain unclear. Research suggests that this disease may be associated with allergic reactions triggered by infections from parasites, viruses, fungi, or insect bites (12). It is hypothesized that an unidentified antigen stimulates mast cells to release IgE, which could be a critical mechanism in its pathogenesis (8). Treatment options for KD include surgical excision, glucocorticoids, local radiotherapy, cytotoxic drugs, biologics, and combination therapies, but the optimal approach remains controversial (13). Here, we report a case of KD with concurrent atopic dermatitis, diagnosed at Shenzhen University General Hospital in Shenzhen, China.

## 2 Case presentation

**Figure 1:**
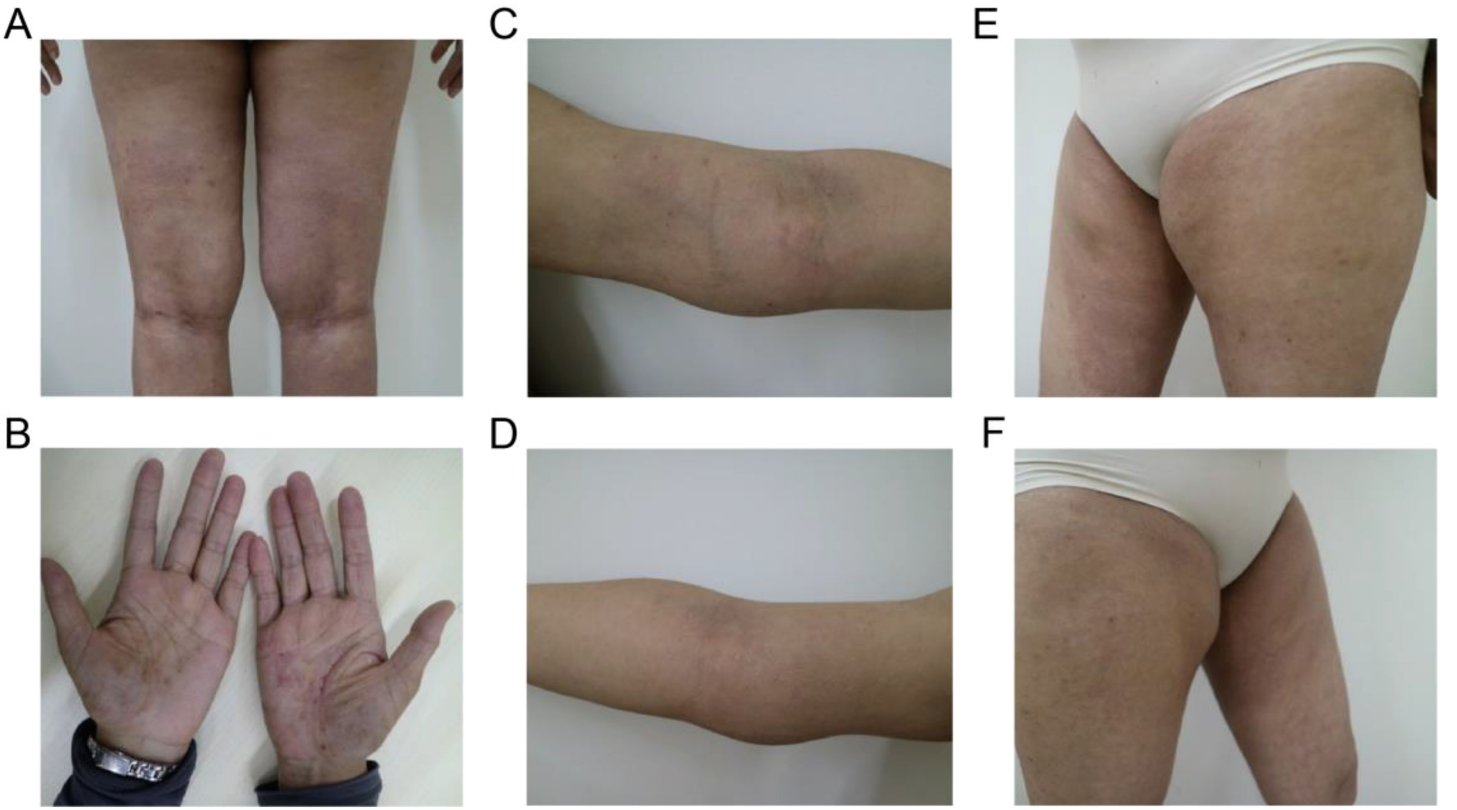
Typical clinical manifestations observed in the patient before the combined treatment included **(A, B)** erythema and pruritic papules on the lower limbs and palms, **(C, D)** palpable masses on the medial side of the elbows, and **(E, F)** in the bilateral groin area.

A 37-year-old Chinese female presented with a 12-year history of systemic erythema and pruritic papules, accompanied by palpable masses on the inner sides of her limbs for the past three years. Physical examination revealed scattered erythema and small papules with ill-defined borders on the waist and limbs (**Figures 1A, B**). Lichenification was observed in the popliteal fossae and antecubital fossae. Firm, cystic-solid masses with ill-defined borders and smooth surfaces, showing good mobility, were palpable on the inner sides of both elbows (**Figures 1C, D**) and groins (**Figures 1E, F**). Additionally, multiple medium-textured lymph nodes, ranging in size from a pea to a ping-pong ball, were palpable preauricularly, postauricularly, anteriorly and posteriorly in the cervical region, on the left shoulder and back, axillary thoracic wall, and groins, with good mobility and smooth surfaces. Routine blood tests (**Table 1**) revealed a marked elevation in eosinophil levels to 6.5 × 10^9/L, accounting for 56.3% of the total white blood cells, which was 10.67 × 10^9/L. Eosinophils have increased to 14.4 times the normal upper limit (0.45 × 10^9/L). Additionally, lymphocytes accounted for 17.1% of the total. Specifically, lymphocyte subset analysis (**Table 1**) indicated a marked reduction in the proportion of CD3+ T cells, which comprised 36.23% (784.96 cells/μL), compared to the normal lower limit of 50%, representing a 13.77% decrease. Among these, CD3+CD4+ T cells accounted for 18.48% (400.41 cells/μL), (normal lower limit: 27%), representing a 14.33% decrease, and CD3+CD8+ T cells constituted 12.67% (274.43 cells/μL), (normal lower limit: 15%), representing a 2.33% decrease. The CD4+/CD8+ ratio was 1.46 (normal range: 0.7-2.8), indicating a slightly reduced ratio within normal limits. Furthermore, there was a substantial increase in CD16+CD56+ NK cells, which made up 49.36% of the lymphocytes (1069.52 cells/μL), significantly higher than the normal upper limit of 40%, representing a 9.36% increase. Immunological testing (**Table 1**) revealed significantly elevated serum levels of IgE exceeding 50,000 IU/mL (normal range: <100 IU/mL), indicating a 500-fold increase. IgG levels were 20.23 g/L (normal upper limit: 16 g/L), representing a 26.4% increase, and IgM was 2.63 g/L (normal upper limit: 2.3 g/L), indicating a 14.3% increase. Serum cytokine levels were also markedly increased, including interleukin (IL)-5 at 4.37 pg/mL (normal range: <4.15 pg/mL, 5.3% increase), IL-1β at 5.56 pg/mL (normal range: <3.40 pg/mL, 63.5% increase), IL-10 at 6.78 pg/mL (normal range: <4.5 pg/mL, 50.7% increase), and IL-17A at 9.10 pg/mL (normal range: <4.74 pg/mL, 92% increase). Additionally, tests for anti-cardiolipin antibodies, anti-β2-glycoprotein 1 antibodies, serum immunofixation electrophoresis, ENA panel, and ANCA panel revealed no notable findings. The patient had normal liver and renal function tests, and there were no indications of hepatitis B, hepatitis C, syphilis, or HIV. Bone marrow biopsy indicated hypercellular bone marrow with active proliferation of granulocytic, erythroid, and megakaryocytic lineages, accompanied by eosinophilia. Genetic testing results showed no abnormalities in BCR-ABL1 typing and quantitative analysis, no PDGFRα gene rearrangement, and no abnormalities in T-cell and B-cell clonality analysis. Comprehensive CD series testing for acute and chronic leukemia, non-Hodgkin lymphoma (NHL), and myelodysplastic syndromes (MDS) also revealed no abnormalities.

**Table 1:** Laboratory test report.

Parameter	Value	Reference Range	Result
Eosinophil, count	6.5 × 10^9/L	0.02-0.45	Increased
Eosinophil, %	56.3	1.0-6.0	Increased
CD3+ T cells, cells/ul	784.96	603-2990	Normal
CD3+ T cells, %	36.23	50-84	Decreased
CD3+CD4+ T cells, cells/ul	400.41	441-2156	Decreased
CD3+CD4+ T cells, %	18.48	27-51	Decreased
CD3+CD8+ T cells, cells/ul	274.43	125-1312	Normal
CD3+CD8+ T cells, %	12.67	15-44	Decreased
CD4+/CD8+ ratio	1.46	0.7-2.8	Normal
CD16+CD56+ NK cells, cells/ul	1069.52	95-640	Increased
CD16+CD56+ NK cells, %	49.36	7-40	Increased
IgE, IU/mL	>5000	0-100	Increased
IgG, g/L	20.23	7-16	Increased
IgM, g/L	2.63	0.4-2.3	Increased
IL-5, pg/mL	4.37	≤4.15	Increased
IL-1β, pg/mL	5.56	≤3.40	Increased
IL-10, pg/mL	6.78	≤4.50	Increased
IL-17A, pg/mL	9.10	≤4.74	Increased

IL, interleukin.

Ultrasound examination revealed the presence of masses in both the submandibular and left shoulder regions, exhibiting mixed echogenicity suggestive of lymphatic vessel dilatation, with dimensions of approximately 22×23×7 mm and 25×23×5 mm, respectively. Abnormal echogenicity and enlargement were observed in the lymph nodes anterior and posterior to both ears, as well as in the cervical and elbow regions. Additionally, enlarged lymph nodes presenting as abnormal echogenic masses were detected in the bilateral inguinal regions, measuring approximately 45×12×24 mm on the left and 46×27×11 mm on the right. MRI further identified an abnormal signal lesion adjacent to the medial side of the right humerus, measuring approximately 43×17×64 mm, which is likely indicative of an enlarged lymph node. PET-CT findings demonstrated multiple nodular lesions in the submental triangle, bilateral preauricular regions, bilateral submandibular triangles, bilateral carotid triangles, bilateral posterior cervical triangles, bilateral axillary regions, subcutaneous left shoulder region, bilateral elbow regions, bilateral external iliac vessels, bilateral inguinal regions, and proximal thighs, with mildly increased radiotracer uptake and a maximum standardized uptake value (SUVmax) of approximately 5.0.

**Figure 2:**
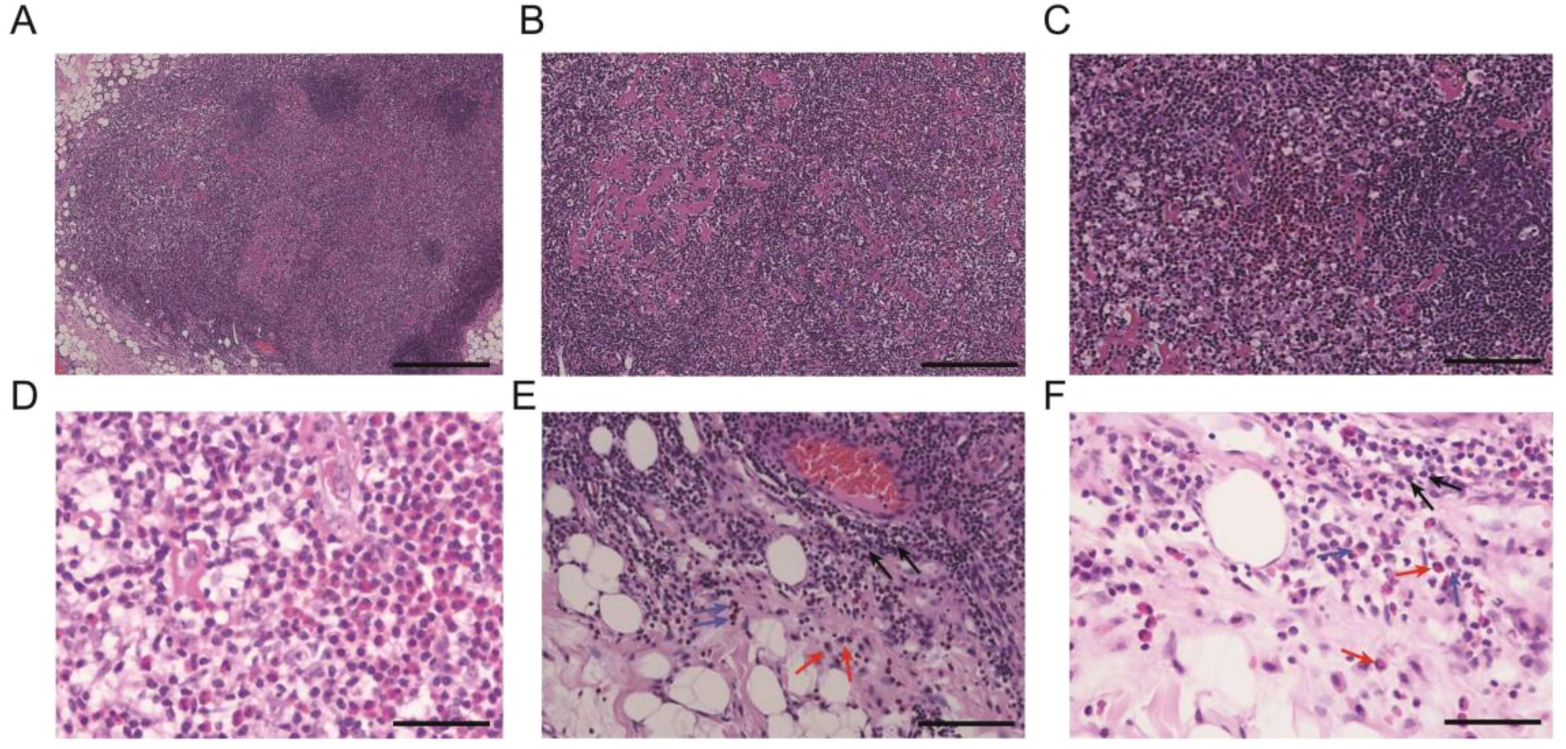
Representational pathological characteristics of masses: **(A)** Reactive lymphoid follicular hyperplasia. **(B)** Stromal fibrosis and hyalinization forming sclerotic stroma. **(C, D)** Formation of eosinophilic microabscesses. **(E, F)** Presence of lymphocytes, eosinophils, and plasma cells in the perinodal fibroadipose tissue. Black arrows: lymphocytes, red arrows: eosinophils, and blue arrows: plasma cells. Scale bars: 250 mm.

**Figure 3:**
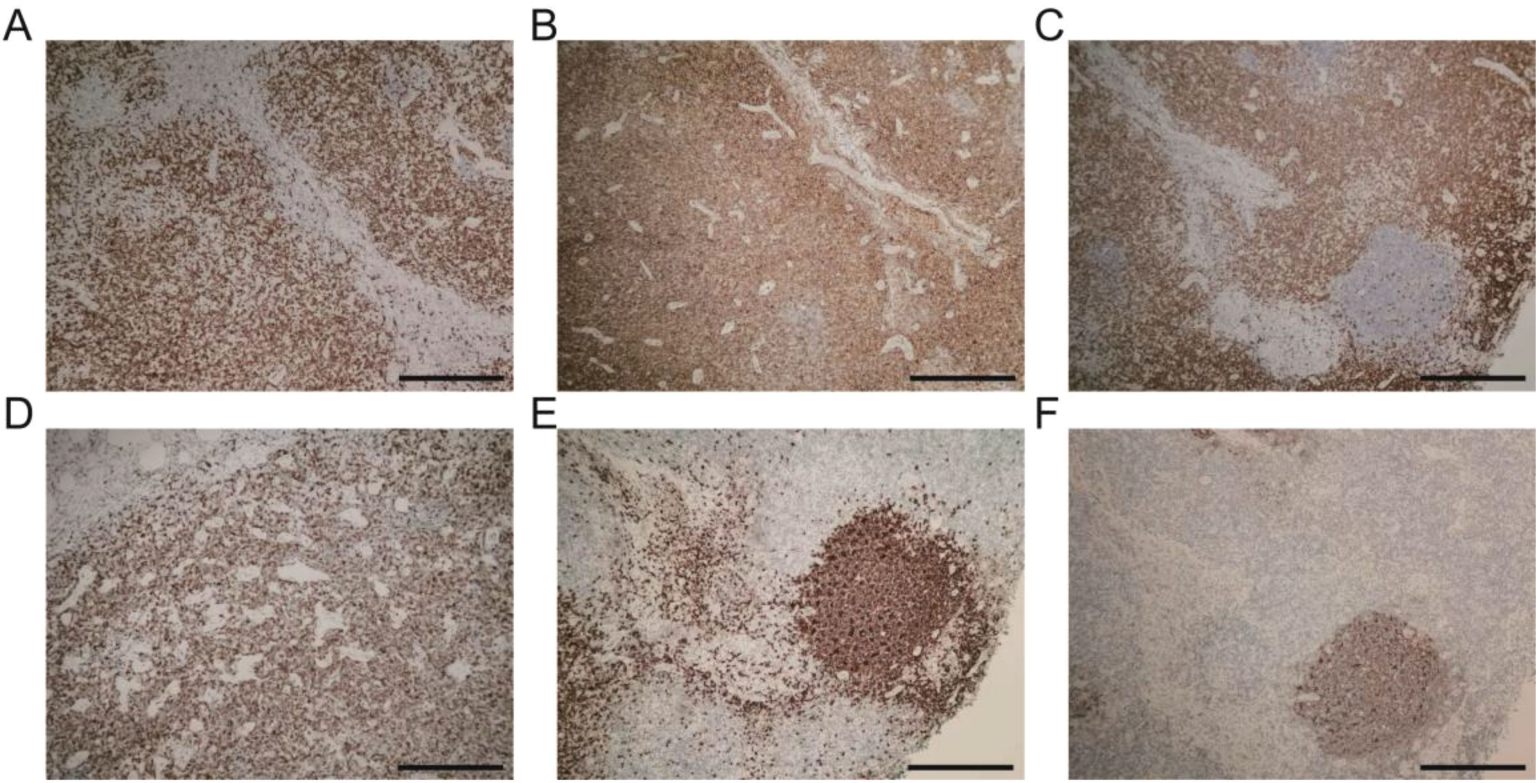
Immunohistochemistry landscape of masses: **(A)** CD3+ positive cells. **(B)** CD4+ positive cells. **(C)** CD5+ positive cells. **(D)** CD7+ positive cells. **(E)** CD20+ positive cells. **(F)** CD21+ positive cells. Scale bars: 250 mm.

The pathological examination (**Figure 2**) of the submental mass and the left cervical lymph node revealed an absence of lymphoid follicles and a distinct capsule (**Figure 2A**). Localized areas containing Langerhans cells were observed. The proliferative fibroadipose tissue (**Figure 2B**) demonstrated extensive infiltration by lymphoplasmacytic cells, histiocytes, and a significant number of eosinophils, accompanied by eosinophilic proteinaceous deposits (**Figures 2C–F**). Furthermore, immunostaining was positive for CD3+ (**Figure 3A**), CD4+ (**Figure 3B**), CD5+ (**Figure 3C**), CD7+ (**Figure 3D**), CD20+ (**Figure 3E**), and CD21+ cells (**Figure 3F**). Collectively, these histopathological findings are consistent with KD.

**Figure 4:**
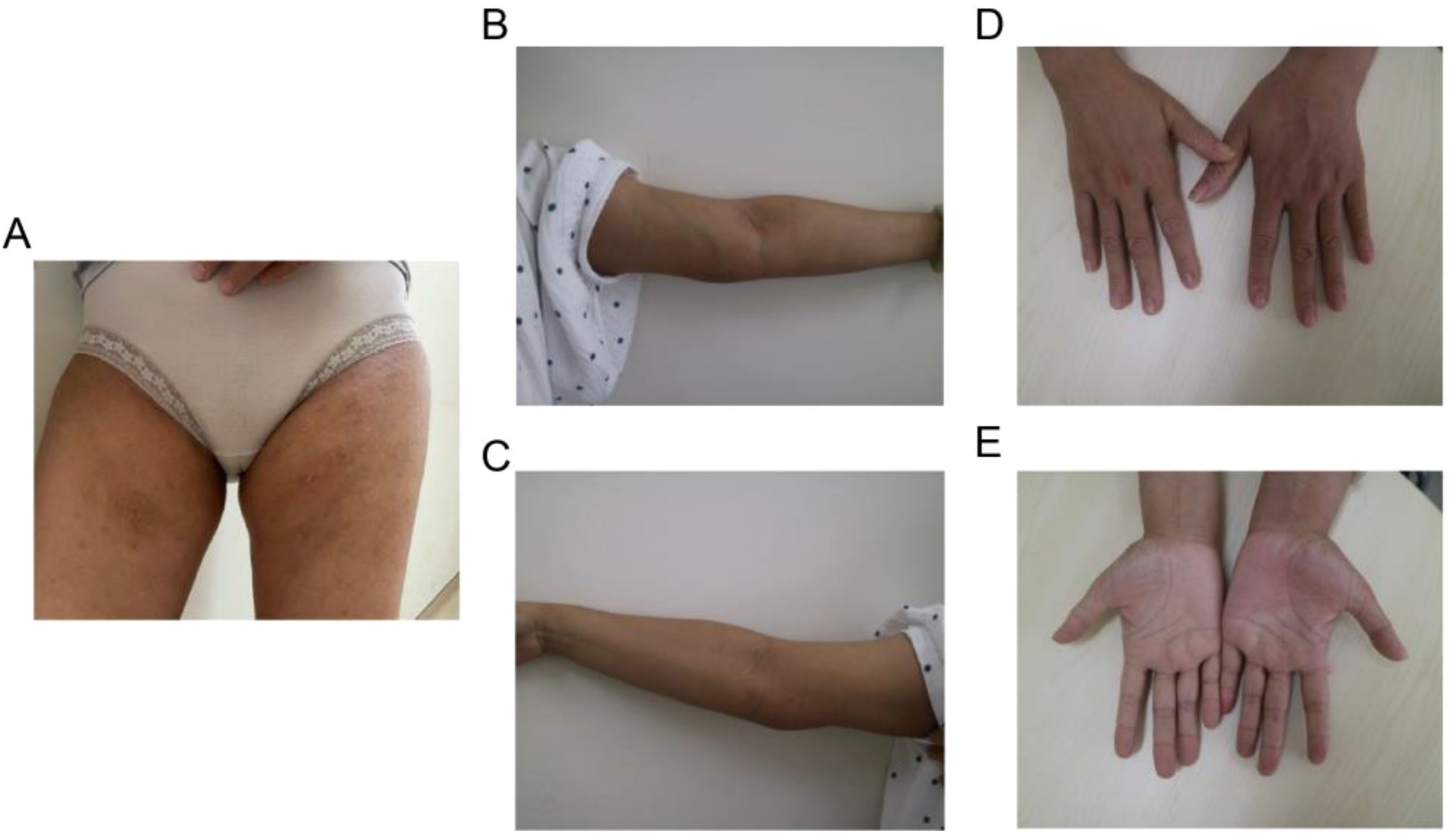
Typical clinical characteristics observed in the patient following combined treatment include shrinkage of masses in **(A)** the bilateral groin area and **(B, C)** the medial side of the elbow, as well as **(D, E)** the fading of erythema and pruritic papules on the palms.

**Figure 5:**
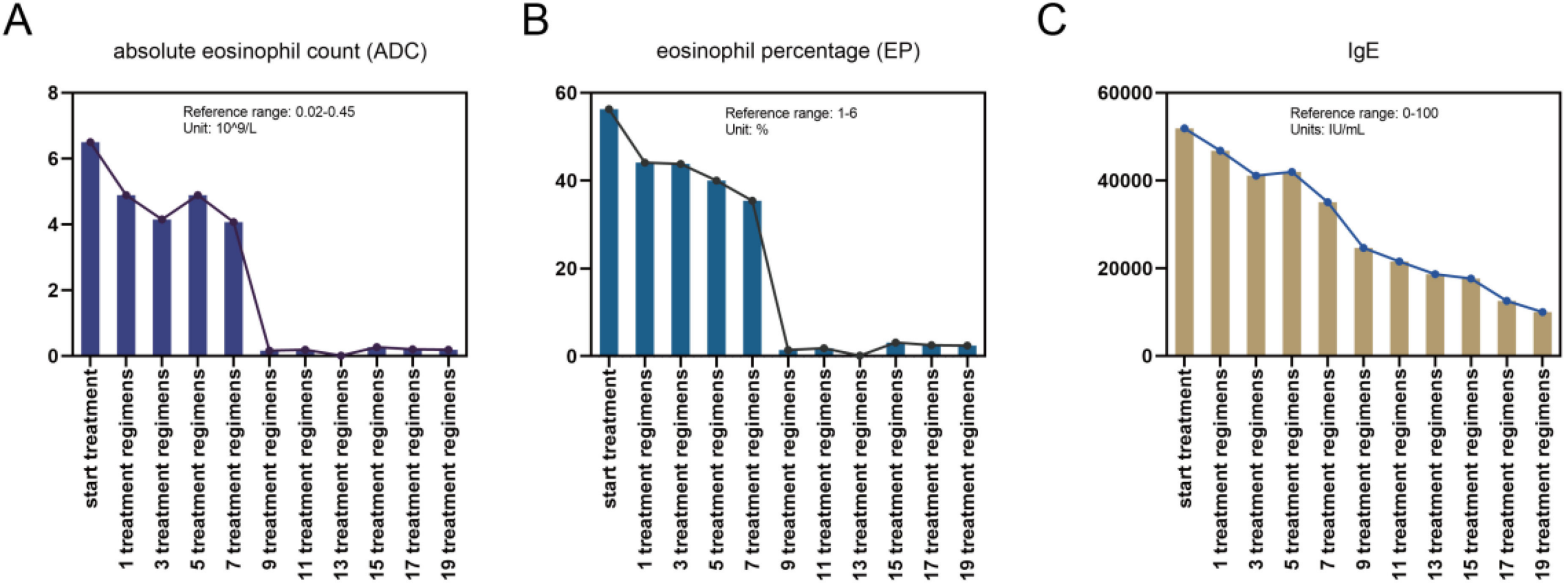
Changes in the **(A)** counts and **(B)** proportions of eosinophils and **(C)** IgE levels during the combined treatment process.

The patient met the diagnostic criteria for KD. Given the presence of multiple enlarged lymph nodes and limb masses, a comprehensive surgical excision is technically challenging, costly, and time-consuming. Considering the absence of contraindications, dupilumab treatment was administered. The treatment regimen consisted of an initial dose of 600 mg (administered as two 300 mg injections), followed by 300 mg every two weeks for a duration of four months via subcutaneous injection. Simultaneously, combined oral corticosteroid therapy (methylprednisolone, initial dose: 16 mg/kg per day, gradually tapered as the tumor regresses). Following the treatment, the patient experienced no severe adverse reactions. A marked reduction in the size of the multiple masses was observed (**Figures 4A–E**). Additionally, the number (**Figure 5A**) and percentage (**Figure 5B**) of eosinophils, as well as serum IgE levels (**Figure 5C**), decreased significantly. The treatment plan and follow-up are ongoing.

## 3 Conclusion

KD, also referred to as eosinophilic granuloma, is a rare, slowly progressing benign inflammatory disorder (13). This disease predominantly affects young Asian men, with a higher prevalence reported in China and Japan, although sporadic cases have been documented in non-Asian populations as well (14). Currently, there are no standardized diagnostic criteria for KD, with diagnosis primarily relying on histopathological examination (15).

Clinically, KD is characterized by single or multiple subcutaneous nodules in the head or neck region, often accompanied by pruritic skin lesions. These subcutaneous nodules and masses are typically painless and are most commonly found in the parotid and submandibular regions. The lesions are frequently associated with regional lymphadenopathy and can be misdiagnosed as parotid tumors due to the involvement of the parotid gland. Other affected sites include the oral cavity, axilla, groin, extremities, and trunk. Systemic symptoms such as fever, night sweats, and weight loss are rare (16, 17).

In our patient, we initially observed a significant increase in both the percentage and count of peripheral blood eosinophils, alongside a marked elevation in serum IgE levels. This required differentiation from conditions such as hypereosinophilia, parasitic infections, and allergic reactions (18). Studies suggest that the immune system plays a crucial role in the pathogenesis of KD (19). In KD patients, elevated levels of IgE, type 2 helper T (Th2) cell cytokines (such as interleukin (IL)-4, IL-5, and IL-13), and thymic stromal lymphopoietin (TSLP) have been documented (20, 21).

Radiological examinations, including ultrasound, CT, and MRI, are valuable for determining the location and size of KD-related masses and are useful for planning subsequent surgical interventions. However, the characteristic imaging features of KD masses have not been clearly defined. Histological examination remains the gold standard for diagnosing KD (15). The histological features of KD in lymph nodes or masses include lymphoid follicular hyperplasia with enlarged germinal centers, eosinophil infiltration, eosinophilic microabscess formation, and postcapillary venule proliferation with circumferential collagen deposition and varying degrees of fibrosis (5).

In this case, initial bone marrow aspiration biopsy revealed active hyperplasia of neutrophils, erythrocytes, and macrophages, along with eosinophilia. Chromosomal analysis showed no abnormalities, effectively ruling out hematological malignancies. Subsequent tissue biopsies of the patient’s submental mass and cervical lymph nodes demonstrated extensive eosinophil infiltration and partial deposition of eosinophilic proteinaceous material. These findings helped exclude lymphoma and superficial tumors, strongly suggesting a diagnosis of KD. Furthermore, we assessed common systemic involvements in KD and found no renal abnormalities, indicating no renal involvement in this patient.

The optimal treatment for KD remains unclear. Current therapeutic options include surgical excision, localized radiation therapy, steroid treatment, and biologic agents (13). Advances in understanding the immunopathogenesis of KD and the development of new biologics have significantly improved treatment strategies. Dupilumab, a fully human-derived monoclonal antibody, specifically binds to IL-4Rα, inhibiting IL-4 and IL-13 signaling (22). Since IL-4 and IL-13 can induce IgE antibody production, a study by Liu et al. demonstrated a significant reduction in serum total IgE levels in six KD patients treated with dupilumab (23). Moreover, research indicates that dupilumab effectively reduces eosinophil counts and percentages in Th2-inflammatory diseases characterized by eosinophil infiltration or elevated serum eosinophil levels (24), potentially benefiting KD patients.

In this case, the patient exhibited multiple lymph node enlargements and limb masses, making complete surgical resection difficult due to high costs and lengthy recovery times. Given the patient’s history of atopic dermatitis, we chose dupilumab for treatment after thorough consideration.

In summary, we report a case of a patient with classic symptoms of KD. We integrated clinical presentation, laboratory tests, imaging, and pathological examinations to exclude other potential conditions and ultimately confirmed a diagnosis of KD. Based on the patient’s condition, we recommend dupilumab for treatment. Due to the limited follow-up data on dupilumab’s effectiveness, further observation is necessary to confirm its therapeutic outcomes.

## Data Availability

The original contributions presented in the study are included in the article/supplementary material. Further inquiries can be directed to the corresponding author.
